# A Prospective Randomized Trial Comparing Radioguided Occult Lesion Localization (ROLL) and Magnetic Seed Localization for the Localization of Nonpalpable Breast Lesions: Analysis of Surgical Outcomes, Patient’s Perception, and Costs

**DOI:** 10.1245/s10434-026-19587-0

**Published:** 2026-04-05

**Authors:** Fabio Corsi, Matilde Pelizzola, Daniela Bossi, Valentina Zanella, Silvana Quaglini, Federico Sottotetti, Sara Albasini, Carlo Morasso

**Affiliations:** 1 Department of Biomedical and Clinical Sciences, University of Milan, Milan, Italy; 2 Breast Unit, Istituti Clinici Scientifici Maugeri IRCCS, Pavia, Italy; 3 General Surgery Residency Program, University of Milan, Milan, Italy; 4 Department of Electrical, Computer and Biomedical Engineering, University of Pavia, Pavia, Italy; 5 Medical Oncology Unit, Istituti Clinici Scientifici Maugeri IRCCS, Pavia, Italy; 6 Laboratory of Nanomedicine, Istituti Clinici Scientifici Maugeri IRCCS, Pavia, Italy

**Keywords:** Breast-conserving surgery, Non-palpable breast lesions, Radioguided occult lesion localization, Magnetic seed localization, Patient-reported outcomes, Randomized trial, Surgical outcomes

## Abstract

**Backgroud:**

Accurate localization of nonpalpable breast lesions in breast-conserving surgery (BCS) is essential for achieving oncological radicality, preserving cosmetic outcomes, and reducing reinterventions for positive margins. Radioguided occult lesion localization (ROLL) and magnetic seed localization (MSL) are established alternatives to wire-guided methods, but comparative evidence remains limited.

**Methods:**

This prospective, single-centre, randomized clinical trial evaluated the noninferiority of MSL compared with ROLL in achieving negative surgical margins, assessed surgical, organizational, and patient-reported outcomes, and included a costs analysis. A total of 260 women with nonpalpable breast lesions suitable for BCS were enrolled between June 2023 and July 2025 and randomized 1:1 to ROLL or MSL. The primary endpoint was margin negativity. Secondary endpoints included calculated resection ratio (CRR), operative time, complications, hospital stay, reoperation rate, and EQ-5D-5L patient-reported outcomes.

**Results:**

Negative margins were achieved in 97.7% of ROLL and 93.1% of MSL cases (*p* = 0.14), confirming MSL noninferiority. Median CRR was identical (1.7, *p* = 0.61). Overall complication rates were comparable (6.2%-MSL vs. 4.7%-ROLL, *p* = 0.59), although postlocalization hematoma was more frequent with MSL (17% vs. 7%, *p* = 0.01). Hospital stay was significantly shorter with MSL (1 vs. 2 days, *p* = 0.001). Operative times were similar, and surgeon experience did not influence margin status, although attendings achieved lower CRR with ROLL. EQ-5D-5L scores were comparable except for “usual activities” dimension, which favoured ROLL.

**Conclusions:**

MSL was noninferior to ROLL, with comparable safety and patient-reported outcomes. Magnetic seed localization was found to be costs-saving across all analysed subgroups. Our results show that MSL is an effective alternative for lesion localization in BCS.

**Supplementary Information:**

The online version contains supplementary material available at 10.1245/s10434-026-19587-0.

Breast-conserving surgery (BCS) is currently the standard treatment approach for nonpalpable breast lesions, aiming to achieve complete tumour excision while preserving the aesthetic integrity of the breast,^1^ as also reaffirmed by the recent consensus session of the St. Gallen International Breast Cancer Conference.^2^ A key determinant of surgical success is the status of the histological margins: positive margins significantly increase the risk of local recurrence, often requiring reoperation.^1,3^ This can negatively affect cosmetic outcomes, delay adjuvant therapies, cause psychological distress, and contribute to increased healthcare costs.^4^ In recent years, the widespread implementation of mammographic screening and advances in imaging techniques have led to a growing detection of small, early-stage, nonpalpable breast lesions, making accurate intraoperative localization an increasingly frequent and critical challenge in breast-conserving surgery.^1,5^

The surgical management of nonpalpable breast lesions presents additional challenges, primarily related to accurate intraoperative localization and complete excision with negative margins.^3^ Various localization techniques are available for clinically nonpalpable lesions.^6^ Traditionally, wire-guided localization (WGL) has been the most commonly used technique for intraoperative identification of nonpalpable breast lesions.^7^ While effective, inexpensive and without any particular technological needs, WGL presents notable limitations, including the potential displacement or fracture of the wire, patient discomfort, and logistical challenges related to same-day placement, which can complicate surgical scheduling.^8^ In response to these limitations, several alternative localization strategies have been developed to improve surgical precision, patient experience, and organizational efficiency.^7^ Various localization techniques are now available for clinically occult lesions. Some of these techniques are now widely implemented in clinical practice, whereas others, such as radar-based systems like SAVI SCOUT^®^, are beginning to be adopted in several breast units.^9^ Among the established techniques radioguided occult lesion localization (ROLL) and magnetic seed localization (MSL) have gained widespread use in breast units, including Istituti Clinici Scientifici Maugeri (ICSM) institution. These findings are consistent with the results of a broader 2023 survey conducted by Senonetwork, which identified the most commonly adopted localization techniques across Italian Breast Units.^10^

Both ROLL and MSL are considered effective and reliable for localizing nonpalpable breast tumours, with several clinical studies demonstrating their safety and efficacy.^11–13^ However, the current literature lacks direct comparative studies exclusively evaluating these two techniques. In ICSM’s clinical practice, both techniques are routinely employed, and their use is not currently guided by lesion characteristics or patient factors.

This registered (ClinicalTrials.gov Identifier: NCT05942118), prospective, randomized study aimed to assess the noninferiority of MSL compared with ROLL in localization of nonpalpable breast lesion achieving negative surgical margins. Secondary endpoints included comparison for operative times, localization’s procedure-related complications, postoperative complications, reoperation rates, and patient’s satisfaction. Finally, a comparison was also made between experienced surgeons and surgeons in training.

## Patients and Methods

### Study Design

This study was conducted at the Breast Unit of the ICSM in Pavia, Italy (institute’s ethical approval: CE 2778, ClinicalTrials.gov Identifier: NCT05942118). It involved female patients with nonpalpable breast lesions identified through imaging and with a confirmed preoperative histological or cytological diagnosis of B3, B4, B5 or C3, C4, C5, who were candidates for BCS. All patients meeting the inclusion criteria provided written informed consent prior to inclusion in the study. Participants were consecutively and randomly assigned to one of two study groups: nonpalpable breast lesions localized using either ROLL (group 1) or MSL (group 2). In ROLL,^14,15^ under ultrasound guidance or, in the case of microcalcifications not visible on ultrasound, under stereotactic guidance, the radiotracer is injected directly into the lesion via a percutaneous approach. The injection is generally performed on the morning of surgery or on the preceding day, depending on operating room scheduling and radiotracer availability within the Nuclear Medicine Department, which is not available before 11:00 a.m. The procedure lasts only a few minutes when performed under ultrasound guidance and approximately 15 min when performed under stereotactic guidance. During surgery, the surgeon is guided for resection by an audible and numerical signal emitted by the probe. Conversely, the MSL^16,17^ procedure involved percutaneous placement of the clip into the lesion after skin disinfection and administration of local anaesthetic. Magseed localization was performed under ultrasound or stereotactic. During surgery, an audible and numerical signal from the SentiMag probe provides real-time feedback to the surgeon on the distance between the marker and the probe, as well as the strength of the magnetic field, to precisely locate the nonpalpable lesion for removal. At the conclusion of the surgical procedure, an x-ray on the surgical specimen was performed, directly in the operating room, to confirm the presence of the clip in the surgical specimen.

In the present study, the lesions were nonpalpable, with a mean size of 9 mm; therefore, also owing to accurate preoperative localization, oncoplastic surgery was not required in any case. Simple direct excision for lesions of this volume ensured an excellent cosmetic outcome.

At ICSM, both techniques have been routinely employed for several years, and all surgeons had extensive, consolidated experience with both approaches. For each patient, clinical and pathological data were collected, including age, menopausal status, body mass index (BMI), type of surgery, surgeon who performed surgery (attendant or resident), and lesion characteristics, such as size, imaging features, and histological or cytological classification.

The primary endpoint of the study was the rate of negative surgical margins, defined according to the widely accepted “no ink on tumour” criterion for invasive carcinoma and a 2-mm margin for *in situ* carcinoma.^18^ Secondary endpoints included the calculated resection ratio (CRR),^19^ which is a quantitative indicator used to assess the adequacy of tissue excision during BCS (it represents the ratio between the volume of the excised specimen and the volume of the target lesion; further explanations in Supplementary Fig. S1), as well as operative time, length of hospital stay, the rate of complications both pre- and postoperatively, and rate of reoperations. Additional analyses were performed according to the operating surgeon’s level of experience to assess whether one localization technique was more suitable than the other for residents or attending surgeons.

### Patient-Reported Outcomes

The study also evaluated patient-reported outcomes (PROs) as an additional secondary endpoint. For this purpose, during their postoperative multidisciplinary follow-up visit, all enrolled patients were asked to complete the EQ-5D-5L questionnaire, referring specifically to their postoperative period. The EQ-5D-5L is a standardized, generic, preference-based instrument for measuring health-related quality of life, developed by the EuroQol Group (https://euroqol.org/).^20,21^ It comprises two components: the descriptive system and the Visual Analogue Scale (EQ VAS). The descriptive system includes five dimensions—mobility, self-care, usual activities, pain/discomfort, and anxiety/depression—each with five response levels: no problems, slight problems, moderate problems, severe problems, and extreme problems. Respondents select the statement that best describes their condition for each dimension. The responses generate a five-digit code representing the individual’s health profile. This code is converted into a so-called index value (or utility value), i.e., a coefficient ranging between 0 and 1, representing the perceived desirability of the health state. The conversion is performed using a mathematical model tailored to the specific population to which the patient belongs.^22^ Specifically, the Italian EQ-5D-5L value set, derived by using the EuroQol Valuation Technology (EQ-VT) protocol and provided by EuroQol Group, was applied to calculate the index for the enrolled patients.^21,23^

The EQ VAS assesses self-rated health on a vertical visual analogue scale ranging from “The worst health you can imagine” (0) to “The best health you can imagine” (100), providing an additional quantitative measure of patient-perceived quality of life.

### Patient Population and Randomization Groups

All consecutive patients referred to the Breast Unit of ICSM in Pavia from June 2023 to July 2025, presenting with a clinically nonpalpable breast lesion on imaging and an indication for BCS, were included in the study after obtaining written consent. Inclusion criteria were nonpalpable breast lesion (microcalcifications, solid nodules, or parenchymal distortions) requiring intraoperative localization, indication for breast-conserving surgery, preoperative histological diagnosis of B3, B4, or B5, or cytological diagnosis of C3, C4, or C5. Patients who underwent neoadjuvant therapy were excluded from the study. In the MSL group, lesion localization was performed using a paramagnetic seed placed under ultrasound guidance. In the ROLL group, localization was achieved through injection of Technetium-99m within 24 hr before surgery. The sentinel lymph node was identified using two main techniques: lymphoscintigraphy (LS) and indocyanine green (ICG). In most cases, patients localized with ROLL underwent sentinel lymph node biopsy (SLNB) with LS (96.3%), whereas those localized with MSL underwent SLNB with ICG (96.1%). However, the two methods are inherently interchangeable.^24^ All radiological and surgical procedures were performed according to standard clinical practice, and surgical specimens were submitted for definitive histopathological examination to assess margin status.

### Costs Analysis for Localization Procedures

The costs analysis was conducted within comparable groups: 1) patients who underwent breast-conserving surgery combined with SLNB, and 2) patients who underwent only breast surgical resection. To estimate surgical costs in the selected subgroups and across the two localization techniques, a bottom-up micro-costing approach was applied.^25^ Only direct costs that differed between the clinical pathways following each procedure were identified and included in the analysis. Personnel costs were not considered incremental, because the clinical workload does not meaningfully differ between the two techniques, apart from a minimal time variation of only a few minutes. Similarly, depreciation related to the intraoperative probes (gamma and magnetic) used for ROLL and MSL were considered comparable and therefore not treated as differential costs. The analysis included the cost per hour of operating room time and the cost per hospitalization day, as defined by the administrative office of ICSM, weighted according to the median operative times (in minutes) and length of stay (in days) observed in the present study. For ROLL, the identified direct costs comprised lymphoscintigraphy, technetium-99m vials, and ROLL-guided ultrasound. For MSL, direct costs included localization ultrasound, the magnetic seed, and indocyanine green vials used for sentinel lymph node detection.

### Statistical Analysis

Randomization was performed by using the National Cancer Institute Clinical Trial Randomization Tool (https://ctrandomization.cancer.gov), applying asymptotic maximal randomization for allocation sequence generation, with an arm assignment ratio of 1:1.^26^ The randomization sequence calculated and applied in the study is detailed in Supplementary Table S1 of supplementary material. Each study arm included 130 patients, for a total enrolment of 260 patients. The randomization list was held by the ICSM Breast Unit’s data manager. Once the surgeon identified and enrolled a patient, after obtaining written informed consent, they contacted the data manager by phone to determine the patient’s assigned intervention group.

Variables were presented as medians, interquartile ranges and ranges (median (interquartile range) [range]), or as absolute numbers and percentages. Categorical variables were compared by using the χ^2^ test or Fisher's exact test, as appropriate, while continuous variables were compared by using the Student’s *t* test or the Wilcoxon two-sample test, following assessment of normality with the Kolmogorov–Smirnov test. Statistical significance was defined as *p* < 0.05 (two-tailed). Data analysis was performed by using SAS software (v. 9.4, SAS Institute Inc., Cary, NC).

## Results

### Baseline Characteristics of Groups

**Fig. 1:**
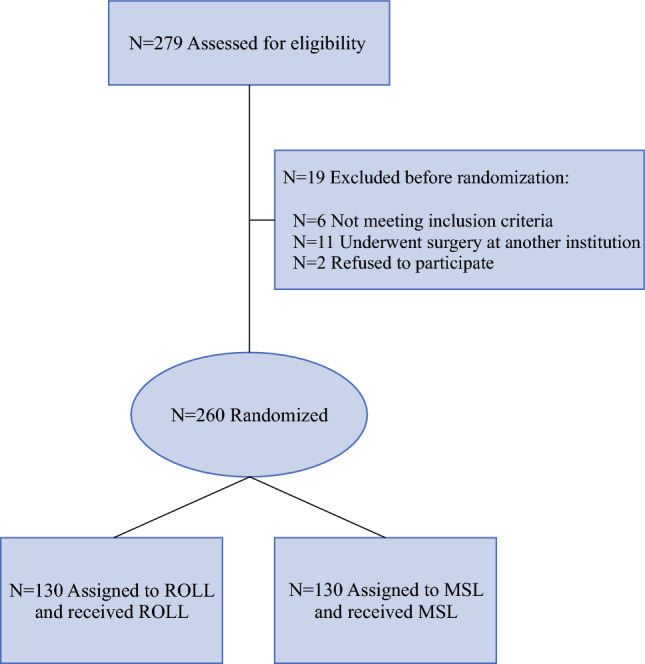
Study profile

Between June 2023 and July 2025, 279 patients with confirmed nonpalpable breast lesions candidates for surgery were initially considered eligible for inclusion. However, 19 patients were excluded prior to randomization: six did no more meet the inclusion criteria at the time of enrolment, 11 underwent surgery at another institution, and two declined to participate (Fig. 1). As shown in Table 1, a total of 260 patients were ultimately randomized in this prospective, single-centre randomized study: 130 underwent MSL and 130 underwent ROLL. Given the randomized design of the trial, the two groups were well balanced across almost all reported variables. The only significant differences concerned the sentinel lymph node detection technique and the use of surgical specimen X-ray (both *p* < 0.0001). Patients in the ROLL group predominantly underwent SLNB using LS (96.3%), whereas those in the MSL group underwent SLNB using IG (96.1%). In addition, surgical specimen X-ray was required in the MSL group to confirm the presence of the magnetic seed. The majority of lesions were malignant (B5b/C5, 85.4% of total), occurring in 112 cases (86.1%) in the ROLL group and 110 cases (84.6%) in the MSL group. For B3/C3 and B4/C4 lesions, there were a total of 28, corresponding to 10.8% of the total. Of these, 14 were in the seed group and 14 in the ROLL group. In this subset of lesions, we observed an upgrade to malignant lesions in 32.1% of cases (Supplementary Table S2).

**Table 1:** Baseline characteristics of the entire cohort

Characteristics	ROLL n = 130	MSL n = 130	*p*
Age at diagnosis (yesr)*	65 (16) [36–87]	66 (19) [38–89]	0.26
Hormonal status			
Fertile	22 (17.3)	20 (15)	0.69
Menopause	105 (82.7)	109 (85)	
Patients' body mass index (BMI)*	25 (6) [18–46]	25 (6) [17–43]	0.95
Lesion size on imaging (mm)*	9 (5) [3–23]	9 (5) [4–35]	0.36
Previous breast lesion			
No	107 (83.6)	114 (87.7)	0.35
Yes	21 (16.4)	16 (12.3)	
Lesion morphology			
Nodule	123 (94.6)	118 (90.8)	0.42
Microcalcification	2 (1.5)	5 (3.9)	
Distorsion	5 (3.9)	7 (5.4)	
Cyto/histological result (CB)			
B3/C3/ B4/C4	14 (10.8)	14 (10.8)	0.81
B5a	4 (3.1)	6 (4.6)	
B5b/C5	112 (86.1)	110 (84.6)	
Surgeon who performed resection			
Attending surgeon	103 (84.4)	109 (87.2)	0.53
Resident	19 (15.7)	16 (12.8)	
Surgical radicalization			
No	13 (10)	18 (13.8)	0.34
Yes	117 (90)	112 (86.2)	
Type of axillary surgery			
None	20 (15.6)	29 (22.3)	0.35
SLNB only	104 (81.3)	96 (73.8)	
ALND	4 (3.1)	5 (3.9)	
SLN detection technique			
LS	104 (96.3)	4 (3.9)	<0.0001
ICG	4 (3.7)	98 (96.1)	
Surgical specimen’s X-ray			
No	122 (94.6)	18 (15.9)	<0.0001
Yes	7 (5.4)	111 (86.1)	
Surgical specimen dimensions (mm)			
X*	50 (20) [13–115]	50 (20) [20–85]	0.66
Y*	37 (15) [2–110]	40 (15) [2–80]	0.88
Z*	25 (15) [5–117]	25 (15) [6–90]	0.92
Surgical specimen weight (g)*	17 (18) [2–274]	15 (18) [2–79]	0.39
Final lesion size (mm)*	10 (6) [2–35]	10 (8) [2–30]	0.51
pT			
No (benign lesion)	13 (10)	12 (9.2)	0.57
pTIS	5 (3.9)	10 (7.7)	
pT1mic	0 (0)	1 (0.8)	
pT1	107 (82.3)	103 (79.2)	
pT2	5 (3.9)	4 (3.1)	

Values are n (%) unless otherwise indicated; values * are median (interquartile range) [range]

*MSL* magnetic seed localization; *ROLL* radioguided occult lesion localization; *CB* core biopsy; *SLNB* sentinel lymph-node biopsy; *SLN* sentinel lymph-node; *ALND* axillary lymph-nodes dissection; *LS* lymphoscintigraphy; *ICB* fluorescence with indocyanine green

For B3 lesions, until the end of 2025 our centre adopted a relatively aggressive approach, favouring breast-conserving surgery, as documented in a recently published study by our group.^27^ This strategy was justified by the uncertainty about the true rate of upgrade of atypical ductal hyperplasia (ADH) to ductal carcinoma in situ or invasive carcinoma. As a matter of fact, it is highly variable and difficult to predict, with reported rates ranging from 5 to 50%.^28^ Regarding papillary lesions, all cases in our series were associated with ADH. As reported by Kulka et al.,^29^ surgical excision is indicated in papilloma associated with ADH, although the relatively low risk of upgrade may allow avoidance of surgical excision in selected cases after multidisciplinary team discussion. All our cases were discussed and shared within a multidisciplinary meeting.

Since the end of 2025, our centre progressively modified patient management strategy towards a less invasive approach, in accordance to the released of the EUSOMA 2024 guidelines,^28^ which recommend surgical excision for ADH lesions larger than 15 mm and vacuum-assisted excision for smaller lesions.

### Postlocalization Procedure and Surgical Outcomes

As shown in Table 2, regarding the margin involvement, there was no statistical difference between ROLL and MSL (respectively free margins for 97.7% vs. 93.1%, *p* = 0.14). This finding remained consistent with the number of reinterventions performed for positive surgical margins (respectively 2.3% vs. 6.9%, *p* = 0.14) and when patients were stratified by BMI: as presented in Supplementary Table S3, among patients with BMI < 30, no significant difference in margin involvement between two groups was observed (*p* = 0.08). No cases of surgical margin involvement were observed among patients with BMI ≥ 30.

**Table 2:** Outcomes under investigation

Characteristics	ROLL n = 130	MSL n = 130	*p*
Surgical margins			
Not involved	127 (97.7)	121 (93.1)	0.14
Involved	3 (2.3)	9 (6.9)	
Reinterventions			
No	125 (97.7)	121 (93.1)	0.14
Yes	3 (2.3)	9 (6.9)	
Postlocalization procedure hematoma			
No	119 (93)	107 (83)	0.01
Yes	9 (7)	22 (17)	
Surgery time (min)			
BCS-only*	34 (20) [15-75]	40 (10) [10-65]	0.14
BCS with SLNB*	51 (16) [30–120]	55 (20) [30–140]	0.27
BCS with ALND*	80 (22) [60–105]	95 (8) [75–140]	0.39
CRR*	1.7 (2) [1–60]	1.7 (2) [1–11]	0.61
Surgical complications			
No	123 (95.3)	122 (93.8)	0.59
Yes	6 (4.7)	8 (6.2)	
Hospitalization (days)*	2 (1) [1–7]	1 (1) [1–4]	0.001

Values are n (%) unless otherwise indicated; values * are median (interquartile range) [range]

*MSL* magnetic seed localization; *ROLL* radioguided occult lesion localization; *BCS* breast-conserving surgery; *SLNB* sentinel lymph-node biopsy; *ALND* axillary lymph-nodes dissection; *CRR* calculated resection ratio

A postlocalization procedure hematoma occurred more frequently in the MSL group compared with ROLL (17% vs. 7%, *p* = 0.01). Surgical time (minutes) did not show statistically significant differences, but there was a trend towards longer duration for MSL respect to ROLL in all subgroups: BCS-only (respectively 40 (10) [10–65] vs. 34 (20) [15–75], *p* = 0.14); BCS with SLNB (respectively 55 (20) [30–140] vs. 51 (16) [30–120], *p* = 0.27); and BCS with ALND (respectively 95 (8) [75–140] vs. 80 (22) [60–105], *p* = 0.39). was identical in median between groups (1.7 (2) [1–60] for ROLL and 1.7 (2) [1–11] for MSL, *p* = 0.61), and surgical complications were observed in 6.2% of patients for MSL and 4.7% for ROLL without statistical difference (*p* = 0.59). Hospitalization days were longer in the ROLL group compared with MSL (2 (1) [1–7] vs. 1 (1) [1–4], *p* = 0.001).

As shown in Supplementary Table S4, all study outcomes were re-evaluated after excluding B3 and B4 lesions from the analyses. The results obtained from this restricted sample were fully consistent with those previously reported, with no meaningful differences observed in postlocalization procedure and surgical outcomes.

### Evaluation of Surgical Outcomes According to Localization Technique and Operating Surgeon

The analysis for comparing surgeries performed by residents and attending surgeons was reported in Table 3. For both localization procedures, there was no difference in margin involvement between attending surgeons and residents (margins free for ROLL: respectively 98.1% vs. 100%, *p* = 1.00. For MSL: respectively 93.6% vs. 87.5%, *p* = 0.32). Attendings achieved significantly better CRR using the ROLL procedure, with a median of 1.5 (2.2) [1–60] compared with 3.3 (2.9) [1–10] for residents (*p =* 0.01). In contrast, no statistically significant difference was observed in the MSL group between attendings and residents, both showing low values (respectively 1.6 (2) [1–11] vs. 1.5 (1) [1–6], *p* = 0.56).

**Table 3:** Evaluation of surgical outcomes according to localization technique and operating surgeon

	ROLL			MSL		
	Attending surgeon	Resident	*p*	Attending surgeon	Resident	*p*
Surgical margins						
Not involved	101 (98.1%)	19 (100%)	1.00	102 (93.6%)	14 (87.5%)	0.32
Involved	2 (1.9%)	0 (0%)		7 (6.4%)	2 (12.5%)	
CRR*	1.5 (2.2) [1–60]	3.3 (2.9) [1–10]	0.01	1.6 (2) [1–11]	1.5 (1) [1–6]	0.56
Surgery time (min)						
BCS-only*	35 (25) [15–75]	30 (14) [20–34]	0.38	42 (12) [10–65]	38 (15) [27–50]	0.61
BCS with SLNB*	51 (18) [30–120]	55 (12) [38–83]	0.45	55 (20) [30–140]	57 (15) [47–85]	0.32
BCS with ALND*	80 (45) [60–105]	80 (0) [80–80]	1.00	95 (8) [75–140]	–	N/A
Surgical complications						
No	98 (96.1%)	17 (89.5%)	0.24	101 (92.7%)	16 (100%)	0.59
Yes	4 (3.9%)	2 (10.5%)		8 (7.3%)	0 (0%)	

Values are n (%) unless otherwise indicated; values * are median (interquartile range) [range]

*MSL* magnetic seed localization; *ROLL* radioguided occult lesion localization; *BCS* breast-conserving surgery; *SLNB* sentinel lymph-node biopsy; *ALND* axillary lymph-nodes dissection

Regarding operative time, there were no statistically significant differences between types of surgeon in any localization group. For BCS-only, median operative times were 35 (25) [15–75] min for attendings and 30 (14) [20–34] min for residents in the ROLL group (*p* = 0.38), and 42 (12) [10–65] minutes versus 38 (15) [27–50] minutes in the MSL group (*p* = 0.61). Similar results were observed for BCS with SLNB, with no significant differences between attendings and residents in either localization method (for ROLL *p* = 0.45 and for MSL *p* = 0.32). For BCS with ALND, operative times were comparable within the ROLL group (median 80 minutes for both; *p* = 1.00).

Postoperative complications were infrequent and comparable between attendings and residents in both groups. In the ROLL cohort, complications occurred in 3.9% of attending-performed procedures and 10.5% of resident-performed procedures (*p* = 0.24), while in the MSL group, complications occurred in 7.3% of patients among attendings and 0% among residents (*p* = 0.59).

### Patient-Reported Outcomes Measures

The results of the patient-reported outcomes are reported in Table 4. No substantial differences were observed between the two localization methods across almost all questionnaire domains (mobility *p* = 0.07, self-care *p* = 0.13, pain/discomfort *p* = 0.62, anxiety/depression *p* = 0.40, and self-perception of health today *p* = 0.43). The only statistically significant finding concerned usual activities: compared with the MSL group, a greater proportion of patients in the ROLL group reported no functional limitations in daily activities, accompanied by a lower rate of patients reporting slight limitations (no limitations: 79% for ROLL vs. 66% for MSL; slight limitations: 15% for ROLL vs. 26% for MSL, *p* = 0.01). The EQ-VAS, that is a global evaluation of the current quality-of-life, did not show any significant differences (*p* = 0.43).

**Table 4:** Patient-reported outcome measures with EQ-5D-5L questionnaire results

	Mobility			Self-care			Usual activities			Pain/discomfort			Anxiety/depression		
	ROLL	MSL	*p*	ROLL	MSL	*p*	ROLL	MSL	*p*	ROLL	MSL	*p*	ROLL	MSL	*p*
1	113 (89)	105 (85)	0.07	105 (82)	89 (72)	0.13	101 (79)	81 (66)	0.01	73 (57)	78 (63)	0.62	77 (61)	61 (49)	0.40
2	10 (8)	12 (10)		19 (15)	25 (20)		19 (15)	32 (26)		40 (32)	34 (28)		29 (23)	39 (32)	
3	1 (1)	6 (5)		2 (2)	8 (7)		4 (3)	10 (8)		14 (11)	11 (9)		16 (12)	15 (12)	
4	3 (2)	0 (0)		1 (1)	1 (1)		3 (3)	0 (0)		0 (0)	0 (0)		4 (3)	6 (5)	
5	0 (0)	0 (0)		0 (0)	0 (0)		0 (0)	0 (0)		0 (0)	0 (0)		1 (1)	2 (2)	

Self-perception of health today*		
ROLL	MSL	*p*
80 (20) [20–100]	75 (15) [30–100]	0.43

Values are n (%) unless otherwise indicated; values * are median (interquartile range) [range]

*MSL* magnetic seed localization; *ROLL* radioguided occult lesion localization

### Cost and Quality of Life Analysis of MSL Compared with ROLL

The EQ-5D index values were calculated to summarize the individual patient heath profiles. In the BCS-only subgroup, the median quality-of-life index was 0.98 (0.12) [0.36–1] in the ROLL group and 0.96 (0.14) [0.68–1] in the MSL group (*p* = 0.86). As shown in Table 5, the total differential cost per procedure per patient was €1520.98 for ROLL and €1245.98 for MSL.

**Table 5:** Costs analysis based on institutional cost data provided by Istituti Clinici Scientifici Maugeri (Pavia, Italy) and data observed in the study

BCS-only Differential costs per surgery							
ROLL Cost components	Unit cost (€)	Quantity	Cost per procedure (€)	MSL Cost components	Unit cost (€)	Quantity	Cost per procedure (€)
Operating room (cost/h)	770	34 min	436.33	Operating room (cost per hour)	770	40 min	513.33
Hospitalization (cost/day)	400	2 days	800	Hospitalization (cost per day)	400	1 day	400
Lymphoscintigraphy	157	1	157	Localization ultrasound	42.65	1	42.65
Technetium-99m (1 vial)	85	1	85	Magnetic seed (1 piece)	290	1	290
ROLL ultrasound	42.65	1	42.65				
Total			**1520.98**				**1245.98**

BCS with SLNB Differential costs per surgery							
ROLL Cost components	Unit cost (€)	Quantity	Cost per procedure (€)	MSL Cost components	Unit cost (€)	Quantity	Cost per procedure (€)
Operating room (cost/h)	770	51 min	654.50	Operating room (cost per hour)	770	55 min	705.83
Hospitalization (cost/day)	400	2 days	800	Hospitalization (cost per day)	400	1 day	400
Lymphoscintigraphy	157	1	157	Localization ultrasound	42.65	1	42.65
Technetium-99m (1 vial)	85	2	170	Magnetic seed (1 piece)	290	1	290
ROLL ultrasound	42.65	1	42.65	Indocyanine green (1 vial)	166.40	1/2	83.2
Total			**1824.15**				**1521.68**

Bold indicates the total costs for each procedure and different surgical setting

*ROLL* radioguided occult lesion localization; *MSL* magnetic seed; *BCS* breast-conserving surgery; *SLNB* sentinel lymph-node biopsy

In the BCS with SLNB subgroup, the median index value was 0.95 (0.11) [0.43–1] for the ROLL group and 0.91 (0.11) [0.42–1] for the MSL group (*p* = 0.02). The corresponding total cost per procedure per patient was €1,824.15 for ROLL and €1,521.68 for MSL.

## Discussion

This prospective randomized study compared MSL with ROLL for the surgical management of nonpalpable breast lesions. To date, there are no direct prospective comparative studies between ROLL and MSL, whereas most available evidence compares each technique separately with WGL.^30,31^ The results of this study demonstrated that MSL was noninferior to ROLL in achieving negative surgical margins, and no significant differences in complication rates were found.

The absence of statistically significant differences in lesion size, BMI, or histological distribution between groups indicates an appropriate balance between randomization arms. The similar CRR values observed in both groups further confirm the comparable surgical precision achieved by both localization methods. Although the ROLL group showed a slightly higher rate of negative margins, the difference was not statistically significant, corroborating the noninferiority hypothesis and the reproducibility of MSL as a standard-of-care localization method as seen also in other studies.^32,33^

We observed a higher incidence of postlocalization hematoma in the MSL group (17%): these data might be partially explained by the mechanical nature of the magnetic seed insertion. Previous large-scale evaluations of magnetic localization have reported similar minor pre- and postoperative complication rates, emphasizing the satisfactory safety profile of MSL in clinical practice.^34–36^ In all cases, the hematoma did not require specific treatment and did not result in delays or modifications to the surgical plan.

ROLL requires coordination between nuclear medicine and surgery, posing potential challenges in institutions with limited radiopharmaceutical facilities or tight operating lists. From a practical standpoint, this logistical independence represents one of the key advantages of magnetic localization, particularly in high-volume breast units or during periods of resource constraint.

Hospitalization stay was longer for ROLL (2 vs. 1 day, *p* = 0.001), a finding that likely reflects differences in perioperative organization rather than in the techniques themselves. In ICSM, the workflow for MSL enables seed placement during pre-admission, allowing surgery to occur on the same day of hospitalization. In contrast, patients undergoing ROLL are frequently admitted the day before surgery, mainly due to logistical constraints associated with radiotracer preparation and nuclear medicine scheduling. In this study, length of hospital stay was calculated as the number of days during which the patient was actually hospitalized, regardless of whether admission occurred on the day before surgery or on the same day. A minority of patients (particularly those undergoing ROLL) were admitted prior to surgery, generally on the day preceding the procedure to allow for radiotracer injection and preoperative lymphoscintigraphy. In selected cases, particularly for B3 lesions, localization could be performed on the same day as surgery without overnight hospitalization. Nevertheless, at our center, BCS combined with SLNB is not routinely performed on a day-surgery basis, which affects overall hospitalization duration.

A relevant finding emerging from our data is that no increase in surgical complications or reoperations was observed in the MSL group, supporting its reliability and reproducibility.

Our data show that the rate of margin involvement did not differ significantly between attendings and residents for either localization technique, suggesting that both ROLL and MSL are reliable methods even when performed by less experienced surgeons under supervision. Interestingly, attending surgeons achieved significantly better CRR, with respect to residents, when using the ROLL technique, indicating that ROLL may be more operator-dependent and that surgical expertise could enhance its precision. In contrast, no difference in CRR was observed between attendings and residents when using MSL, possibly reflecting the greater procedural standardization and reproducibility of the magnetic seed method. Operative time did not vary significantly between surgeon levels in either localization technique, regardless of whether BCS was performed alone or combined with SLNB or ALND. These findings suggest that both localization procedures are well integrated into surgical workflows and can be performed efficiently by surgeons at different stages of training. Postoperative complications were infrequent and comparable between groups, further supporting the safety and reproducibility of both ROLL and MSL.

From a patient-centred perspective, patient-reported outcomes revealed no significant differences between the two localization methods across most assessed domains, including mobility, self-care, pain, and psychological well-being. However, a higher proportion of patients in the ROLL group reported no limitations in performing daily activities compared with the MSL group, accompanied by a lower rate of slight limitations. This finding may reflect subtle differences in patient comfort, tissue handling, or localization device characteristics, although further studies are warranted to confirm this trend. Notably, the discomfort associated with these procedures is generally minimal and temporary, typically lasting only a few days.^11^

Despite slightly longer operative times, MSL was associated with lower total procedural costs in both surgery subgroups—BCS-only and BCS with SLNB—demonstrating a more favourable cost profile compared with ROLL. Overall savings were primarily driven by shorter hospitalization and the elimination of lymphoscintigraphy and technetium-99m-relatad expenses. In the BCS-only subgroup, MSL was economically dominant, as it was associated with both lower procedural costs and comparable EQ-5D index value respect to ROLL. In BCS with SLNB group, MSL was associated with slightly lower median quality-of-life values compared with ROLL (*p* = 0.02); however, the absolute values remained high (0.91) and close to the perfect health state. Nevertheless, MSL resulted in lower costs per procedure per patient. Taken together, these findings support MSL as a cost-efficient localization strategy.

This study has some limitations that should be addressed with further larger studies: first, its single-centre design may limit the number of enrolled patients and findings could be influenced by institutional practices or workflow organization. Second, despite randomization, most procedures were performed by attending surgeons, and subtle variations in experience or familiarity with the two localization techniques may have influenced surgical parameters. Another limitation of this study is that cosmetic and patient-reported aesthetic outcomes were not evaluated by using purpose-specific patient-reported outcome measures. However, it should be noted that the mean diameter of the lesions included in the study was 9 mm (interquartile range of 5). This size allows for excellent objective and patient-perceived results, with very limited resections and without significant loss of volume, shape, or symmetry. This is also the reason why, for this type of lesion, specific oncoplastic techniques, involving glandular remodelling, are generally not required. On the other hand, the EQ-VAS, which in principle could capture patient concerns related to cosmetic outcomes, did not demonstrate any significant difference.

Overall, taken together, these results suggest that both ROLL and MSL are safe and effective localization techniques for nonpalpable breast lesions that can be successfully adopted by surgeons with varying levels of experience and appear to have minimal impact on patients’ overall perception of well-being. However, MSL introduces additional organizational and patient-centered benefits that may support its broader implementation as a radiation-free and workflow-optimized alternative to radioguided procedures.

## Supplementary Information

Below is the link to the electronic supplementary material.

Supplementary file1 (DOCX 175 KB)
