# Supplementary material for: A Prospective Randomized Trial Comparing Radioguided Occult Lesion Localization (ROLL) and Magnetic Seed Localization for the Localization of Nonpalpable Breast Lesions: Analysis of Surgical Outcomes, Patient’s Perception, and Costs
Source: Ann Surg Oncol. 2026 Apr 5;33(7):6403–13. doi: 10.1245/s10434-026-19587-0 (PMC13242391; doi:10.1245/s10434-026-19587-0)
Supplement: Supplementary file 1 — Supplementary file1 (DOCX 175 KB) [file 10434_2026_19587_MOESM1_ESM.docx]

**A prospective randomized trial comparing Radioguided Occult Lesion Localization (ROLL) and Magnetic Seed Localization for the localization of non-palpable breast lesions: analysis of surgical outcomes, patient’s perception and costs**

Fabio Corsi^1,2^*, MD, Matilde Pelizzola^3^, MsC, Daniela Bossi^2^, MD, Valentina Zanella^3^, MsC, Silvana Quaglini^4^, PhD, Federico Sottotetti^5^, MD, Sara Albasini^2^, MsC, Carlo Morasso^6^, PhD

1. Department of Biomedical and Clinical Sciences, University of Milan, Milan, Italy
2. Breast Unit, Istituti Clinici Scientifici Maugeri IRCCS, Pavia, Italy
3. General Surgery Residency Program, University of Milan, Milan, Italy
4. Department of Electrical, Computer and Biomedical Engineering, University of Pavia, Italy
5. Medical Oncology Unit, Istituti Clinici Scientifici Maugeri IRCCS, Pavia, Italy.
6. Laboratory of Nanomedicine, Istituti Clinici Scientifici Maugeri IRCCS, Pavia, Italy

*Corresponding Author:

Fabio Corsi, MD

Istituti Clinici Scientifici Maugeri IRCCS, Pavia, Italy

Department of Biomedical and Clinical Sciences, University of Milan, Milan, Italy

E-mail: fabio.corsi@icsmaugeri.it - Phone: +39 0382592272

**Supplementary Figure S1** Mathematical calculation of Calculated Resection Ratio (CRR)^1^

**
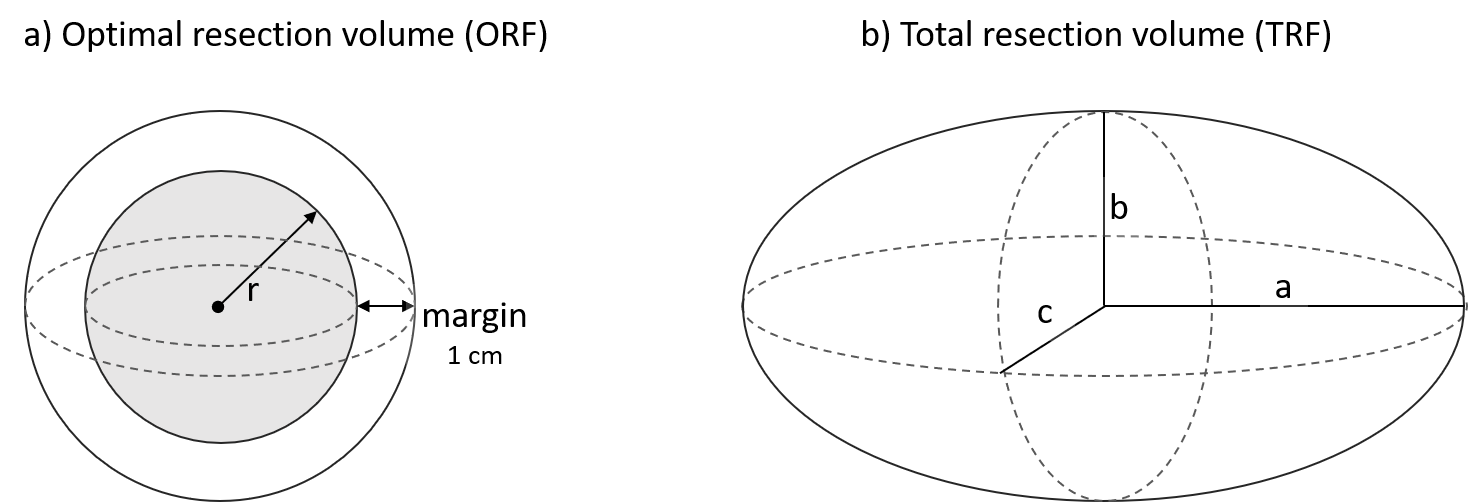
**

The tumour was defined as a sphere, and the tumour volume was calculated by the formula 4/3πr³. *r* represents the radius, which is equal to one half of the diameter measured by the pathologist.

(a) Optimal resection volume (ORV) was calculated for each tumour. ORV was defined as the spherical tumour volume with an added 1.0-cm margin of healthy breast tissue, calculated by the formula 4/3π(*r* + 1.0)³.

(b) Total resection volume (TRV) represents the volume of the surgical specimen. TRV was assumed to be an ellipsoid in shape and was calculated using the three dimensions of the surgical specimen (in cm) measured by the pathologist. The formula applied to calculate TRV was 4/3π(*a·b·c*), with *a*, *b*, and *c* representing one half of each of the three dimensions of the surgical specimen.

CRR was calculated as ratio TRV/ORV.

**Supplementary Table S1** Randomization sequence of the study.

| **Group** | **Subject** | **Assignment** | **Assigned Arm Index** | **Arm 1 Posterior Totals** | **Arm 2 Posterior Totals** |
| --- | --- | --- | --- | --- | --- |
| All | 1 | Roll | 2 | 0 | 1 |
| All | 2 | Roll | 2 | 0 | 2 |
| All | 3 | Magseed | 1 | 1 | 2 |
| All | 4 | Magseed | 1 | 2 | 2 |
| All | 5 | Magseed | 1 | 3 | 2 |
| All | 6 | Roll | 2 | 3 | 3 |
| All | 7 | Roll | 2 | 3 | 4 |
| All | 8 | Magseed | 1 | 4 | 4 |
| All | 9 | Roll | 2 | 4 | 5 |
| All | 10 | Magseed | 1 | 5 | 5 |
| All | 11 | Roll | 2 | 5 | 6 |
| All | 12 | Roll | 2 | 5 | 7 |
| All | 13 | Magseed | 1 | 6 | 7 |
| All | 14 | Roll | 2 | 6 | 8 |
| All | 15 | Magseed | 1 | 7 | 8 |
| All | 16 | Magseed | 1 | 8 | 8 |
| All | 17 | Roll | 2 | 8 | 9 |
| All | 18 | Roll | 2 | 8 | 10 |
| All | 19 | Magseed | 1 | 9 | 10 |
| All | 20 | Roll | 2 | 9 | 11 |
| All | 21 | Magseed | 1 | 10 | 11 |
| All | 22 | Magseed | 1 | 11 | 11 |
| All | 23 | Roll | 2 | 11 | 12 |
| All | 24 | Roll | 2 | 11 | 13 |
| All | 25 | Magseed | 1 | 12 | 13 |
| All | 26 | Magseed | 1 | 13 | 13 |
| All | 27 | Roll | 2 | 13 | 14 |
| All | 28 | Magseed | 1 | 14 | 14 |
| All | 29 | Magseed | 1 | 15 | 14 |
| All | 30 | Roll | 2 | 15 | 15 |
| All | 31 | Roll | 2 | 15 | 16 |
| All | 32 | Magseed | 1 | 16 | 16 |
| All | 33 | Magseed | 1 | 17 | 16 |
| All | 34 | Roll | 2 | 17 | 17 |
| All | 35 | Roll | 2 | 17 | 18 |
| All | 36 | Magseed | 1 | 18 | 18 |
| All | 37 | Roll | 2 | 18 | 19 |
| All | 38 | Magseed | 1 | 19 | 19 |
| All | 39 | Roll | 2 | 19 | 20 |
| All | 40 | Magseed | 1 | 20 | 20 |
| All | 41 | Roll | 2 | 20 | 21 |
| All | 42 | Magseed | 1 | 21 | 21 |
| All | 43 | Roll | 2 | 21 | 22 |
| All | 44 | Magseed | 1 | 22 | 22 |
| All | 45 | Magseed | 1 | 23 | 22 |
| All | 46 | Magseed | 1 | 24 | 22 |
| All | 47 | Roll | 2 | 24 | 23 |
| All | 48 | Roll | 2 | 24 | 24 |
| All | 49 | Magseed | 1 | 25 | 24 |
| All | 50 | Roll | 2 | 25 | 25 |
| All | 51 | Roll | 2 | 25 | 26 |
| All | 52 | Magseed | 1 | 26 | 26 |
| All | 53 | Roll | 2 | 26 | 27 |
| All | 54 | Magseed | 1 | 27 | 27 |
| All | 55 | Magseed | 1 | 28 | 27 |
| All | 56 | Roll | 2 | 28 | 28 |
| All | 57 | Magseed | 1 | 29 | 28 |
| All | 58 | Roll | 2 | 29 | 29 |
| All | 59 | Magseed | 1 | 30 | 29 |
| All | 60 | Roll | 2 | 30 | 30 |
| All | 61 | Magseed | 1 | 31 | 30 |
| All | 62 | Magseed | 1 | 32 | 30 |
| All | 63 | Roll | 2 | 32 | 31 |
| All | 64 | Roll | 2 | 32 | 32 |
| All | 65 | Roll | 2 | 32 | 33 |
| All | 66 | Roll | 2 | 32 | 34 |
| All | 67 | Magseed | 1 | 33 | 34 |
| All | 68 | Magseed | 1 | 34 | 34 |
| All | 69 | Magseed | 1 | 35 | 34 |
| All | 70 | Magseed | 1 | 36 | 34 |
| All | 71 | Roll | 2 | 36 | 35 |
| All | 72 | Roll | 2 | 36 | 36 |
| All | 73 | Roll | 2 | 36 | 37 |
| All | 74 | Magseed | 1 | 37 | 37 |
| All | 75 | Magseed | 1 | 38 | 37 |
| All | 76 | Magseed | 1 | 39 | 37 |
| All | 77 | Roll | 2 | 39 | 38 |
| All | 78 | Magseed | 1 | 40 | 38 |
| All | 79 | Roll | 2 | 40 | 39 |
| All | 80 | Roll | 2 | 40 | 40 |
| All | 81 | Magseed | 1 | 41 | 40 |
| All | 82 | Magseed | 1 | 42 | 40 |
| All | 83 | Roll | 2 | 42 | 41 |
| All | 84 | Roll | 2 | 42 | 42 |
| All | 85 | Magseed | 1 | 43 | 42 |
| All | 86 | Roll | 2 | 43 | 43 |
| All | 87 | Roll | 2 | 43 | 44 |
| All | 88 | Magseed | 1 | 44 | 44 |
| All | 89 | Magseed | 1 | 45 | 44 |
| All | 90 | Roll | 2 | 45 | 45 |
| All | 91 | Roll | 2 | 45 | 46 |
| All | 92 | Magseed | 1 | 46 | 46 |
| All | 93 | Roll | 2 | 46 | 47 |
| All | 94 | Magseed | 1 | 47 | 47 |
| All | 95 | Roll | 2 | 47 | 48 |
| All | 96 | Roll | 2 | 47 | 49 |
| All | 97 | Magseed | 1 | 48 | 49 |
| All | 98 | Roll | 2 | 48 | 50 |
| All | 99 | Magseed | 1 | 49 | 50 |
| All | 100 | Magseed | 1 | 50 | 50 |
| All | 101 | Roll | 2 | 50 | 51 |
| All | 102 | Roll | 2 | 50 | 52 |
| All | 103 | Magseed | 1 | 51 | 52 |
| All | 104 | Magseed | 1 | 52 | 52 |
| All | 105 | Roll | 2 | 52 | 53 |
| All | 106 | Magseed | 1 | 53 | 53 |
| All | 107 | Roll | 2 | 53 | 54 |
| All | 108 | Magseed | 1 | 54 | 54 |
| All | 109 | Roll | 2 | 54 | 55 |
| All | 110 | Magseed | 1 | 55 | 55 |
| All | 111 | Magseed | 1 | 56 | 55 |
| All | 112 | Roll | 2 | 56 | 56 |
| All | 113 | Magseed | 1 | 57 | 56 |
| All | 114 | Roll | 2 | 57 | 57 |
| All | 115 | Magseed | 1 | 58 | 57 |
| All | 116 | Roll | 2 | 58 | 58 |
| All | 117 | Roll | 2 | 58 | 59 |
| All | 118 | Magseed | 1 | 59 | 59 |
| All | 119 | Magseed | 1 | 60 | 59 |
| All | 120 | Roll | 2 | 60 | 60 |
| All | 121 | Roll | 2 | 60 | 61 |
| All | 122 | Magseed | 1 | 61 | 61 |
| All | 123 | Roll | 2 | 61 | 62 |
| All | 124 | Magseed | 1 | 62 | 62 |
| All | 125 | Roll | 2 | 62 | 63 |
| All | 126 | Magseed | 1 | 63 | 63 |
| All | 127 | Roll | 2 | 63 | 64 |
| All | 128 | Roll | 2 | 63 | 65 |
| All | 129 | Magseed | 1 | 64 | 65 |
| All | 130 | Magseed | 1 | 65 | 65 |
| All | 131 | Magseed | 1 | 66 | 65 |
| All | 132 | Magseed | 1 | 67 | 65 |
| All | 133 | Roll | 2 | 67 | 66 |
| All | 134 | Magseed | 1 | 68 | 66 |
| All | 135 | Roll | 2 | 68 | 67 |
| All | 136 | Roll | 2 | 68 | 68 |
| All | 137 | Roll | 2 | 68 | 69 |
| All | 138 | Magseed | 1 | 69 | 69 |
| All | 139 | Roll | 2 | 69 | 70 |
| All | 140 | Magseed | 1 | 70 | 70 |
| All | 141 | Roll | 2 | 70 | 71 |
| All | 142 | Magseed | 1 | 71 | 71 |
| All | 143 | Magseed | 1 | 72 | 71 |
| All | 144 | Roll | 2 | 72 | 72 |
| All | 145 | Roll | 2 | 72 | 73 |
| All | 146 | Magseed | 1 | 73 | 73 |
| All | 147 | Roll | 2 | 73 | 74 |
| All | 148 | Magseed | 1 | 74 | 74 |
| All | 149 | Magseed | 1 | 75 | 74 |
| All | 150 | Magseed | 1 | 76 | 74 |
| All | 151 | Roll | 2 | 76 | 75 |
| All | 152 | Roll | 2 | 76 | 76 |
| All | 153 | Roll | 2 | 76 | 77 |
| All | 154 | Magseed | 1 | 77 | 77 |
| All | 155 | Roll | 2 | 77 | 78 |
| All | 156 | Magseed | 1 | 78 | 78 |
| All | 157 | Magseed | 1 | 79 | 78 |
| All | 158 | Magseed | 1 | 80 | 78 |
| All | 159 | Roll | 2 | 80 | 79 |
| All | 160 | Magseed | 1 | 81 | 79 |
| All | 161 | Roll | 2 | 81 | 80 |
| All | 162 | Magseed | 1 | 82 | 80 |
| All | 163 | Roll | 2 | 82 | 81 |
| All | 164 | Magseed | 1 | 83 | 81 |
| All | 165 | Roll | 2 | 83 | 82 |
| All | 166 | Magseed | 1 | 84 | 82 |
| All | 167 | Roll | 2 | 84 | 83 |
| All | 168 | Magseed | 1 | 85 | 83 |
| All | 169 | Roll | 2 | 85 | 84 |
| All | 170 | Roll | 2 | 85 | 85 |
| All | 171 | Magseed | 1 | 86 | 85 |
| All | 172 | Roll | 2 | 86 | 86 |
| All | 173 | Roll | 2 | 86 | 87 |
| All | 174 | Magseed | 1 | 87 | 87 |
| All | 175 | Roll | 2 | 87 | 88 |
| All | 176 | Magseed | 1 | 88 | 88 |
| All | 177 | Roll | 2 | 88 | 89 |
| All | 178 | Magseed | 1 | 89 | 89 |
| All | 179 | Roll | 2 | 89 | 90 |
| All | 180 | Magseed | 1 | 90 | 90 |
| All | 181 | Magseed | 1 | 91 | 90 |
| All | 182 | Roll | 2 | 91 | 91 |
| All | 183 | Roll | 2 | 91 | 92 |
| All | 184 | Magseed | 1 | 92 | 92 |
| All | 185 | Magseed | 1 | 93 | 92 |
| All | 186 | Roll | 2 | 93 | 93 |
| All | 187 | Roll | 2 | 93 | 94 |
| All | 188 | Roll | 2 | 93 | 95 |
| All | 189 | Magseed | 1 | 94 | 95 |
| All | 190 | Roll | 2 | 94 | 96 |
| All | 191 | Magseed | 1 | 95 | 96 |
| All | 192 | Roll | 2 | 95 | 97 |
| All | 193 | Magseed | 1 | 96 | 97 |
| All | 194 | Magseed | 1 | 97 | 97 |
| All | 195 | Roll | 2 | 97 | 98 |
| All | 196 | Magseed | 1 | 98 | 98 |
| All | 197 | Roll | 2 | 98 | 99 |
| All | 198 | Magseed | 1 | 99 | 99 |
| All | 199 | Magseed | 1 | 100 | 99 |
| All | 200 | Roll | 2 | 100 | 100 |
| All | 201 | Roll | 2 | 100 | 101 |
| All | 202 | Magseed | 1 | 101 | 101 |
| All | 203 | Magseed | 1 | 102 | 101 |
| All | 204 | Roll | 2 | 102 | 102 |
| All | 205 | Magseed | 1 | 103 | 102 |
| All | 206 | Roll | 2 | 103 | 103 |
| All | 207 | Roll | 2 | 103 | 104 |
| All | 208 | Roll | 2 | 103 | 105 |
| All | 209 | Magseed | 1 | 104 | 105 |
| All | 210 | Roll | 2 | 104 | 106 |
| All | 211 | Magseed | 1 | 105 | 106 |
| All | 212 | Roll | 2 | 105 | 107 |
| All | 213 | Magseed | 1 | 106 | 107 |
| All | 214 | Magseed | 1 | 107 | 107 |
| All | 215 | Magseed | 1 | 108 | 107 |
| All | 216 | Roll | 2 | 108 | 108 |
| All | 217 | Magseed | 1 | 109 | 108 |
| All | 218 | Magseed | 1 | 110 | 108 |
| All | 219 | Roll | 2 | 110 | 109 |
| All | 220 | Roll | 2 | 110 | 110 |
| All | 221 | Roll | 2 | 110 | 111 |
| All | 222 | Magseed | 1 | 111 | 111 |
| All | 223 | Roll | 2 | 111 | 112 |
| All | 224 | Magseed | 1 | 112 | 112 |
| All | 225 | Roll | 2 | 112 | 113 |
| All | 226 | Roll | 2 | 112 | 114 |
| All | 227 | Magseed | 1 | 113 | 114 |
| All | 228 | Magseed | 1 | 114 | 114 |
| All | 229 | Roll | 2 | 114 | 115 |
| All | 230 | Magseed | 1 | 115 | 115 |
| All | 231 | Roll | 2 | 115 | 116 |
| All | 232 | Roll | 2 | 115 | 117 |
| All | 233 | Magseed | 1 | 116 | 117 |
| All | 234 | Magseed | 1 | 117 | 117 |
| All | 235 | Magseed | 1 | 118 | 117 |
| All | 236 | Magseed | 1 | 119 | 117 |
| All | 237 | Roll | 2 | 119 | 118 |
| All | 238 | Roll | 2 | 119 | 119 |
| All | 239 | Magseed | 1 | 120 | 119 |
| All | 240 | Roll | 2 | 120 | 120 |
| All | 241 | Roll | 2 | 120 | 121 |
| All | 242 | Roll | 2 | 120 | 122 |
| All | 243 | Magseed | 1 | 121 | 122 |
| All | 244 | Magseed | 1 | 122 | 122 |
| All | 245 | Roll | 2 | 122 | 123 |
| All | 246 | Magseed | 1 | 123 | 123 |
| All | 247 | Magseed | 1 | 124 | 123 |
| All | 248 | Roll | 2 | 124 | 124 |
| All | 249 | Roll | 2 | 124 | 125 |
| All | 250 | Magseed | 1 | 125 | 125 |
| All | 251 | Magseed | 1 | 126 | 125 |
| All | 252 | Roll | 2 | 126 | 126 |
| All | 253 | Roll | 2 | 126 | 127 |
| All | 254 | Magseed | 1 | 127 | 127 |
| All | 255 | Magseed | 1 | 128 | 127 |
| All | 256 | Magseed | 1 | 129 | 127 |
| All | 257 | Roll | 2 | 129 | 128 |
| All | 258 | Roll | 2 | 129 | 129 |
| All | 259 | Magseed | 1 | 130 | 129 |
| All | 260 | Roll | 2 | 130 | 130 |

When the study was designed in 2023, there was a lack of literature data comparing the use of magnetic seeds and the ROLL technique for the localization of non-palpable breast lesions. Therefore, MSL was considered comparable to radioactive seed localization, for which a disease-free surgical margins rate of 93.2% had been reported, compared with 95.9% for ROLL^2^. Assuming a study power of 80%, a two side confidence level of 90%, and a non-inferiority margin of 6.4% for difference between techniques, a total of n=130 patients per study group was calculated as necessary for adequate statistical power^3^.

**Supplementary Table S2** B3/C3 and B4/C4 lesions

| **B3/C3 and B4/C4 lesions** | **Number** | **Size (median)** | **Final histology DCIS** | **Final histology infiltrating** |
| --- | --- | --- | --- | --- |
| Atypical ductal hyperplasia (ADH) | 9 | 8 mm | 2 (22%) | 0 |
| Papillary lesions with ADH | 11 | 10 mm | 0 | 1 (9%) |
| Flat epithelial atypia (FEA) | 1 | 15 mm | 0 | 0 |
| Radial scar | 1 | 6 mm | 0 | 0 |
| B4 at core biopsy | 6 | 9 mm | 2 (33%) | 4 (67%) |

DCIS= Ductal carcinoma in situ

**Supplementary Table S3** Outcomes under investigation with Body Mass Index (BMI) stratification

|  | **BMI <30** | | | **BMI ≥30** | | |
| --- | --- | --- | --- | --- | --- | --- |
|  | **ROLL** | **MSL** | **p** | **ROLL** | **MSL** | **p** |
| **Surgical margins** |  |  |  |  |  |  |
| Not involved | 112 (97.4) | 105 (92.1) | 0.08 | 15 (100) | 17 (100) | N/A |
| Involved | 3 (2.6) | 8 (7.9) |  | 0 (0) | 0 (0) |  |

Values are n (%). BMI= Body Mass Index; ROLL= Radioguided Occult Lesion Localization; MSL= Magnetic seed.

**Supplementary Table S4** Outcomes under investigation excluding B3/B4 lesions at core biopsy

| **Characteristics** | **ROLL**  **n=130** | **MSL**  **n=130** | **p-value** |
| --- | --- | --- | --- |
| **Surgical margins** |  |  |  |
| Not involved | 113 (97.4%) | 108 (93.1%) | 0.21 |
| Involved | 3 (2.6%) | 8 (6.9%) |  |
| **Reinterventions** |  |  |  |
| No | 112 (97.4%) | 109 (93.3%) | 0.33 |
| Yes | 3 (2.6%) | 7 (6.7%) |  |
| **Post-localization procedure hematoma** |  |  |  |
| No | 105 (92.1%) | 95 (82.6%) | 0.03 |
| Yes | 9 (7.9%) | 20 (17.4%) |  |
| **Surgery time (min)** |  |  |  |
| BCS-only* | 43 (23) [30-65] | 43 (15) [25-65] | 0.98 |
| BCS with SLNB* | 50 (15) [30-90] | 55 (20) [30-140] | 0.27 |
| BCS with ALND* | 80 (22) [60-105] | 95 (8) [75-140] | 0.41 |
| **CRR*** | 1.8 (2) [1-60] | 1.8 (2) [1-11] | 0.72 |
| **Surgical complications** |  |  |  |
| No | 109 (94.8%) | 108 (93.1%) | 0.59 |
| Yes | 6 (5.2%) | 8 (6.9%) |  |
| **Hospitalization (days)*** | 2 (2) [1-7] | 1 (1) [1-4] | 0.002 |

Values are n (%) unless otherwise indicated; values * are median (i.q.r.) [range]. MSL= Magnetic seed Localization; ROLL= Radioguided Occult Lesion Localization; SLNB= sentinel lymph-node biopsy; ALND= axillary lymph-nodes dissection; CRR= calculated resection ratio.

**References**

1. Krekel NMA, Zonderhuis BM, Stockmann HBAC, et al. A comparison of three methods for nonpalpable breast cancer excision. *European Journal of Surgical Oncology (EJSO)*. 2011;37(2):109-115. doi:10.1016/j.ejso.2010.12.006

2. Niinikoski L, Hukkinen K, Leidenius MHK, et al. Resection margins and local recurrences of impalpable breast cancer: Comparison between radioguided occult lesion localization (ROLL) and radioactive seed localization (RSL). *The Breast*. 2019;47:93-101. doi:10.1016/j.breast.2019.07.004

3. Chow SC, Shao J, Wang H. *Sample Size Calculations in Clinical Research*. 2nd Ed. Chapman & Hall/CRC
